# Oral health-related quality of life and reasons for discontinuing partial removable dental prosthesis usage: a cross-sectional study with one to seven years of follow-up

**DOI:** 10.1186/s12903-024-04114-y

**Published:** 2024-03-20

**Authors:** Siraphob Techapiroontong, Nareudee Limpuangthip

**Affiliations:** Department of Prosthodontics, Faculty of Dentistry, Chulalongkorn University, 34 Henri-Dunant Road, Pathumwan, Bangkok, 10330 Thailand

**Keywords:** Partial denture, Oral health, Quality of life, Removable dental prosthesis, Denture use

## Abstract

**Background:**

In partial edentulous individuals, a partial removable dental prosthesis (PRDP) is a common dental replacement option to improve oral function and quality of life. However, some patients discontinue using their denture over time. The aim of this study was to determine the prevalence and characteristics of partial edentulous patients who no longer wear their dentures, explore their reasons, and assess their oral health-related quality of life (OHRQoL).

**Methodology:**

This cross-sectional study, conducted at Chulalongkorn University Dental School from 2013 to 2019, involved patients who received PRDP treatment. They were contacted via phone calls and asked about their denture usage. Eligible participants were patients who had stopped or rarely used their PRDPs. Data on oral status, health insurance, and PRDP variables were collected from hospital records. Telephone interviews were conducted to collect the initial reasons for seeking PRDP treatment, reasons for discontinuation, desire for a new PRDP, and OHRQoL. The Oral Impacts on Daily Performances index was used to assess the OHRQoL. The score was dichotomized into the absence or presence of oral impacts. Chi-square tests and multivariable binary logistic regression were employed to determine the associations between oral impacts and various factors in the participants who discontinued PRDP usage.

**Results:**

Among the 975 contacted participants, 175 (17.9%) discontinued using their PRDPs. Most of these individuals had at least 20 remaining natural teeth and/or 4 posterior occluding pairs. The primary initial reason for seeking PRDP treatment was often based on a dentist’s suggestion. Although many participants reported no impact on OHRQoL and did not express the need for new PRDPs, those experiencing oral impacts were more likely to seek replacements.

**Conclusions:**

With up to 7 years follow-up duration, individuals with partial edentulism and sufficient remaining functional dentition without oral impacts were more inclined to discontinue PRDP usage. Those with maxillary anterior teeth loss were less likely to discontinue using their PRDP. The primary initial reason for seeking PRDP treatment was often a dentists’ suggestion. However, the individuals reporting oral impacts expressed their needs for new denture replacements. This highlights the significance of incorporating patient needs and preferences in prosthodontic decision-making.

## Background

Tooth loss is a global oral health concern, along with dental caries and periodontal disease [1]. The absence of teeth can significantly impair an individual’s oral function and oral health-related quality of life (OHRQoL), concurrent with increased malnutrition risk [2, 3]. Presently, the rate of complete edentulism is declining and the prevalence trend is now shifting towards being partially edentulous [4, 5]. However, without adequate oral rehabilitation, the effect of tooth loss could extend beyond oral health, affecting an individual’s general well-being and increasing the risk of comorbidities [2, 6].

After experiencing tooth loss, patients typically undergo prosthodontic treatment to replace the missing teeth and regain lost function. In case of partial edentulism, various treatment modalities have been implemented, either fixed or removable dental prostheses. A partial removable dental prosthesis (PRDP) is one of the treatments of choice because it has been shown to improve the masticatory ability, self-satisfaction, and OHRQoL of individuals with partial edentulism [7–9].

Although PRDPs present a valuable treatment option for patients with partial edentulism [10], some partial edentulous individuals discontinue wearing them on subsequent follow ups [11, 12]. Previous studies have identified factors influencing the continuation of PRDP use after delivery [8, 10–12]. Other studies reported that patients tend to wear their PRDP when it improves their masticatory function or enhances esthetics [13, 14]. Denture base types, such as acrylic-based (APRDP) or metal-based (MPRDP), Kennedy’s classification, and previous denture experience had no impact on discontinuing PRDP usage [11–14]. Moreover, the availability of healthcare insurance could affect the discontinuation rate of PRDP usage, with patients receiving their PRDP for free under universal health coverage are more prone to stop wearing them [15]. In Thailand, an PRDP is a widely provided dental replacement for partial edentulous patients because the treatment cost is partially or totally covered by various national health insurance programs, including the Universal Health Coverage Scheme (UCS), Civil Servant Medical Benefit Scheme (CSMBS), and Social Security Scheme (SSS). The CSMBS is available to civil servants and those who have retired, as well as their parents, spouse, and children. The SSS is available to individuals who are employed in the formal sector, including private-sector and government employees, and certain categories of self-employed individuals, while other individuals are eligible for the UCS.

Despite extensive discussions on the factors related to discontinuation of PRDP usage [10–14], little attention has been given to the initial reasons why patients seek PRDP treatment and the impact on their OHRQoL after discontinuation. To fill the knowledge gap, the primary aim of this study was to determine the prevalence and characteristics of partial edentulous patients who no longer use their PRDPs, and secondarily to explore their characteristics, reasons for not wearing, and assess their OHRQoL status.

## Methodology

### Study design and participants

The present cross-sectional study was conducted from January 2021 to January 2022. We focused on patients with partial edentulism who had undergone PRDP treatment, either metal-based or acrylic-based, from 2013 to 2019 at the undergraduate or postgraduate prosthodontic clinic at Chulalongkorn University Dental School. The patients were contacted via telephone to maximize the response rate, particularly among those who were unable or unwilling to return for maintenance recall appointments. The patients that were contacted were asked for permission to participate in a brief telephone interview, during which they were asked about the frequency of their denture use.

Eligible participants in the study were partial edentulous individuals who had discontinued using either their maxillary or mandibular PRDP or both for at least one year, or who rarely use their PRDP(s) (less than 1–2 days per week). Patients whose PRDP was part of a full-mouth rehabilitation and those who were unwilling to provide personal information or cooperate with the interview were excluded. Ethical approval for the study was obtained from the Human Research Ethics Committee of the Faculty of Dentistry, Chulalongkorn University (HREC-DCU 2020-094).

### Sample size calculations

The sample size was determined using G*Power based on the proportions of two independent groups. A previous follow-up study in partial edentulous individuals found that the usage rate of acrylic-based PRDP and metal-based PRDP 2–4 years after denture delivery were 60.2% and 80.0%, respectively [15]. With a 5% significance level and 80% power, a sample size of 90 per group was calculated. After including the two denture base materials and a 10% non-response rate, the total sample size required in this study was 200.

### Data collection

Information was collected from hospital records and patient telephone interviews. Hospital records provided information on health insurance, oral status, and PRDP. Health insurance categories included the UCS, CSMBS, SSS, company welfare, no insurance, and others. Oral status variables comprised the number of remaining natural teeth (< 20, ≥ 20 NT), the number of posterior occluding pairs (< 4, ≥ 4 POP), and edentulous location. The variables related to PRDP consisted of material type (metal-based or acrylic-based PRDP) and tooth- versus tissue-support (tooth-supported, Kennedy classification III, IV with a < 4 teeth edentulous span; tissue-supported, Kennedy classification I, II, or IV with a ≥ 4 teeth edentulous span).

During the telephone interview, the participants were asked about underlying health issues and denture usage. This consisted of their reasons for initially seeking PRDP treatment, their reasons for discontinuing PRDP usage, and whether there was a need for a new prosthesis (yes, no). Furthermore, the participants were asked to express their OHRQoL based on their current oral and denture conditions.

The OHRQoL was assessed with the Thai-version of Oral Impacts on Daily Performances (OIDP) index [16, 17]. The participants were asked a series of questions to understand how often and how severely they had experienced problems or difficulties related to their oral and dental health in various activities over the past 6 months. The eight activities encompassed: (1) eating, (2) speaking and pronouncing clearly, (3) cleaning teeth and/or denture, (4) sleeping and relaxing, (5) smiling without self-consciousness, (6) maintaining emotional well-being, (7) enjoying social interaction, and (8) carrying out major work. For each of these activities, the participants rated both the frequency and severity of difficulties encountered on a five-point ordinal scale, giving a maximum score of 25 for each activity and the total score ranged from 0 to 200. Subsequently, dummy variables were generated, with 0 signifying no impact on daily activities (OIDP score = 0) and 1 signifying at least one activity (OIDP score > 0).

### Data analysis

The data were analyzed using STATA version 17.0 at a 5% significance level. Descriptive statistics were calculated to report the percent distribution of the participants across related variables. The two-proportion Z-test was used to determine the discontinuation rate difference between the two denture base types. The Chi-square test was employed in the bivariate analysis to assess the association between the presence of oral impacts and individual factors. Then, multivariable binary logistic regression analysis was performed to determine the presence of oral impacts (presence (1), absence (0)) and associated factors in the participants who discontinued using their PRDP, and adjusted odds ratio with confidence intervals were calculated.

## Results

From 2013 to 2019, 1350 patients received PRDP treatment, encompassing both metal-based and acrylic-based PRDPs, with approximately 70% being women (Table 1). Among these, 975 patients (72.2%) were successfully contacted for telephone interviews, in which 586 (60.1%) were female and 389 (39.9%) were male. No significant differences were observed in demographic characteristics (age, sex, health insurance) between those that could and could not be contacted. Among the contacted individuals, 175 participants (17.9%) reported that they had discontinued using their denture.

**Table 1:** Prevalence of the patients who were contactable and discontinued using their PRDP

PRDP type	Total N	Can be contact	Can be contact and discontinue PRDP usage (%)	p-value
APRDP	667	457	76 (16.6)	0.313
MPRDP	683	518	99 (19.1)	

PRDP, partial removable dental prosthesis; APRDP, acrylic-based PRDP; MPRDP, metal-based PRDP

Table 2 presents the characteristics of the participants who discontinued using their PRDPs, categorized based on their patterns of PRDP use. Most of the participants who discontinued PRDP usage were female, between 50 and 69 years old, lacked insurance coverage for their PRDP expenses, and had at least 20 NT and 4 POP. It was noted that the participants with an edentulous area in the anterior region tended to continue using their PRDPs. The most common initial reasons for undergoing PRDP fabrication were a dentist’s suggestion, followed by the desire to enhance their chewing ability and to prevent tooth movement. However, after PRDP delivery, discomfort, food impaction, denture-related pain, and chewing difficulties emerged as the primary reasons for discontinuing of PRDP use. Almost all participants who discontinued using their PRDP (94.8%) did not seek a replacement.

**Table 2:** Characteristics of the participants who discontinued using their PRDP (N=175)

		PRDP wearing status: % by row		
	Overall distribution (100%)	Discontinued wearing both UPRDP and LPRDP (15.0%)	Discontinued wearing only UPRDP (27.8%)	Discontinued wearing only LPRDP (57.2%)
**Age (years)**: <50	13.8			
50–69	72.3			
≥70	13.9			
**Sex**: Male	33.5			
Female	66.5			
**Health insurance**:				
UCS	9.1	73.3	6.7	20.0
Company welfare	0.9	100.0	0.0	0.0
CSMBS	12.4	85.0	5.0	10.0
SSS	8.6	84.6	7.7	7.7
No insurance	68.6	84.4	3.3	12.3
Others	0.4	100.0	0.0	0.0
**Oral status**:				
Remaining natural teeth: < 20	11.0	73.7	5.3	21.0
≥ 20	89.0	85.1	3.9	11.0
Posterior occluding pair: <4	27.8	70.2	2.1	27.7
≥4	72.2	88.8	4.8	6.4
**Edentulous area**:				
Maxillary arch: Anterior only	4.6	0.0	0.0	100.0
Posterior only	41.1	87.4	7.0	5.6
Both (Ant & Post)	12.7	59.1	0.0	40.9
No tooth loss	41.6	100.0	0.0	0.0
Mandibular arch: Anterior only	1.7	100.0	0.0	0.0
Posterior only	80.9	83.6	2.9	13.5
Both (Ant & Post)	8.1	64.3	21.4	14.3
No tooth loss	9.3	100.0	0.0	0.0
**Initial reason for seeking PRDP treatment**:				
Better chewing	17.9	74.2	6.5	19.3
Social activities	0.6	100.0	0.0	0.0
Family suggestion	8.7	85.8	7.1	7.1
Supported by government health coverage scheme	0.6	100.0	0.0	0.0
Replacing old denture	3.5	66.7	0.0	33.3
Dentist suggestion	51.4	85.4	3.4	11.2
Preventing tooth movement	17.3	89.6	3.5	6.9
**Reason for discontinuing PRDP usage**:				
Chewing difficulty	15.6	67.8	7.4	14.8
Food impaction	24.3	90.5	2.4	7.1
Declined taste	2.9	80.0	20.0	0.0
Speaking difficulty	2.3	75.0	25.0	0.0
Ill-fitting denture	3.5	66.7	0.0	33.3
Pain from denture	22.5	87.2	0.0	12.8
Discomfort	26.6	82.6	4.4	13.0
Others i.e. nausea, vomiting	2.3	50.0	0.0	50.0
**Patient’s desire for new PRDP**: No	94.8	83.5	3.7	12.8
Yes	5.2	88.9	11.1	0.0

LPRDP, mandibular partial removable dental prosthesis; UPRDP, maxillary partial removable dental prosthesis; UCS, Universal Healthcare Coverage Scheme; CSMBS; Civil Servant Medical Benefit Scheme; SSS, Social Security Scheme

Oral impacts were reported by 28.9% of the participants who discontinued PRDP usage. The presence of oral impacts was not associated with PRDP wearing or oral status. The participants who reported experiencing at least one oral impact were found to be associated with the desire for a new PRDP by both bivariate and multivariable analyses **(**Table 3**).**

**Table 3:** Prevalence of oral impacts, bivariate, and multivariable analyses of factors associated with discontinuation of PRDP use

	Overall oral impacts		Presence of oral impacts:
	Presence (28.9%)	Absence (71.1%)	Adjusted odds ratio (95% CI)
**Overall**	28.9	71.1	
**Age (years)**: <50	31.3	68.7	1 (ref.)
50–69	31.9	68.1	3.72 (0.96–14.34)
≥70	12.1	87.9	1.55 (0.27–8.89)
**Sex**: Male	30.3	69.7	1 (ref.)
Female	28.4	71.6	0.62 (0.28–1.33)
**PRDP wearing status**:			
Neither UPRDP nor LPRDP	28.3	71.7	1 (ref.)
Not wearing only UPRDP	28.6	71.4	1.81 (0.26–12.33)
Not wearing only LPRDP	38.1	61.9	1.67 (0.57–4.88)
**Initial reason for seeking PRDP treatment**:			
Family suggestion	17.6	82.4	1 (ref.)
Replacing old denture	30.0	70.0	3.67 (0.33–40.12)
Dentist suggestion	27.4	72.6	1.63 (0.32–8.27)
Preventing tooth movement	37.1	62.9	3.39 (0.61–18.89)
Better chewing	33.3	66.7	2.37 (0.41–13.67)
Supported by government health coverage scheme	0.0	100.0	N/A
Social activities	0.0	100.0	N/A
**Oral status**:			
Remaining natural teeth: < 20	11.5	88.5	1 (ref.)
≥ 20	31.5	68.5	2.61 (0.59–11.42)
Posterior occluding pair: <4	25.9	74.1	1 (ref.)
≥4	29.3	70.7	0.76 (0.30–1.93)
**Patients’ need for new PRDP**: No	27.1	72.9	1 (ref.)
Yes	63.6*	36.4	10.66 (1.85–61.39)*

*Significant association at *p* < 0.05

CI, confidence interval; N/A, not applicable due to very low samples in the subgroup (*n* ≤ 2)

## Discussion

To the best of our knowledge, this is the first study to investigate the initial reasons for PRDP treatment in partial edentulous patients who discontinued their usage and to assess their OHRQoL based on their current oral status. The most frequent initial reason for receiving a dental prosthesis in patients who discontinued using their PRDP was their dentist’s suggestion. Interestingly, despite discontinuation, most of the participants did not report any oral impacts or express the need for a new prosthesis. However, among those who did experience oral impacts, there was a notable desire for a new PRDP. Our study further revealed that most of the participants who discontinued using their PRDPs had at least 20 NT, 4 POP, and an edentulous area in a posterior region.

Oral status, including dental status and edentulous location, plays an important role in PRDP use. Partial edentulous participants with at least 20 NT and 4POP demonstrated a tendency to discontinue using their PRDPs. This observation could be because there was minimal impact on their OHRQoL even though there was an edentulous region, aligning with the shortened dental arch concept which stated that satisfaction, function, and OHRQoL could be achieved without dental substitutions [18–22]. Notably, only a few participants who discontinued using their PRDP presented with anterior tooth loss, followed by those losing both anterior and posterior teeth, and lastly, those with only posterior tooth loss. When anterior teeth were lost, the participants tended to wear their upper PRDPs, which are assumed to be more noticeable, than their lower PRDPs. This highlights the impact of maxillary anterior tooth loss on psychological and social disabilities aspects [23]. Consistent with a previous study, our study reinforces the connection between PRDP usage and patients’ satisfaction with esthetics and being pain-free when using the PRDP [24].

The initial reason for PRDP treatment in patients who discontinued using their PRDP were diverse, with the primary reason being a dentist’s suggestion, followed by an expectation of improved chewing ability and preventing of tooth movement. This aligns with the findings of Murai et al. (2015), who stated that one of the reasons that patients discontinued wearing the denture is that they lacked a perceived need for the denture [12]. Given this insight, dentists should carefully screen whether the patients express their PRDP need because of tooth loss that affects their life, or whether it is a professionally assessed need during the history-taking session. If it is the latter, dentists should take time to establish common treatment outcome goals to receive cooperation and enhance treatment compliance [25]. Although patients initially seek treatment to improve their masticatory function and to prevent tooth movement, many discontinue usage after a certain period. This suggests that factors, such as pain and discomfort, might also play an important role on long-term PRDP usage in addition to the initial reasons provided.

Contrasting perspectives on the impact of PRDP usage on the OHRQoL are found in the literature. Shaghaghian et al. (2019) reported that partial edentulous patients who had never or rarely used an PRDP exhibited the worst OHRQoL compared with regular users [26]. This contradicts the findings of a systematic review, which did not observe consistent improvements in OHRQoL among individuals using PRDPs compared with non-users [27]. Our study found that 71.1% of participants who discontinued wearing their dentures did not experience any oral problems that affected their OHRQoL, including in the absence of PRDP usage. However, the presence of oral impacts was significantly associated with the expressed need for a new PRDP. This emphasizes the importance of considering the patient’s OHRQoL when determining the need for a dental prosthesis.

This study revealed that 17.9% of the contacted patients who underwent PRDP treatment at the dental school during the past 7 years discontinued using their PRDP. Notably, there was no significant difference in the discontinuation rates in groups with different denture base materials and tooth- versus tissue-support. Most of the participants in this study paid for the treatment cost themselves, potentially explaining the lower discontinuation rate and the similar usage rates between the two denture base materials. Even though universal health insurance in Thailand covers both denture base materials, whether full coverage or co-payments, financial coverage might not be the primary reason for the lower discontinuation rate. Other factors, such as the presence of remaining natural dentition or the absence of oral impacts, could have more influence. Compared with previous studies that reported the discontinuation rate of PRDP usage ranging from 35 to 40% [11–13, 15], our study stands out with a lower overall rate. This difference may be attributed to financial, oral status, and OHRQoL factors mentioned earlier. Notably, during denture provision, patients were likely to have interacted with other dentists. We would therefore suggest that the lower proportion of patients no longer wearing their denture was unlikely to be due to concerns about upsetting a single denture provider. Even though PRDPs with tooth-support were reported to be more tolerable [28], the association with the discontinuation rate could not be found in the present study, consistent with the previous reports [11, 13].

The multivariable analysis results revealed a significant association only between the need for a new prosthesis and the presence of oral impact. Factors, such as denture wearing and oral status, did not show any significant associations. It could be inferred that most patients who discontinued using their PRDPs predominantly presented with more than 20 NT and 4 POP, mitigating the impact of tooth loss on their OHRQoL. Therefore, the primary consideration when deciding whether a new prosthesis should be made for a patient should be their need for a new prosthesis. In cases where there is no apparent need, sufficient functional dentition, and the minor tooth loss does not affect the OHRQoL, non-intervention could be a more suitable and patient-centered option.

The present study has some limitations. First, there is a potential for recall bias in reporting the severity and frequency on OHRQoL which could happen during telephone interviews, considering it was at least 2 years after PRDP delivery. However, we minimized the bias by dichotomizing the OHRQoL into the presence and absence of impacts. Information regarding PRDP design, which could affect the usage rate [15], was not assessed and discussed in this study. Moreover, as a cross-sectional study, our OHRQoL evaluation could only be collected based on the current situation where the participants had already discontinued using their PRDPs. A more comprehensive understanding could be achieved through a cohort study, enabling the collection of initial OHRQoL status before treatment, thus identifying whether OHRQoL assessments could effectively inform treatment need. The dentists’ reasons for suggesting dental prosthesis treatment to their patients should also be explored to identify the prosthesis need from both perspectives.

These findings have several clinical implications. When patients present with partial edentulism, a comprehensive approach to history-taking and examination is crucial. Attention should be given to their chief complaint and the effects of tooth loss on the patients’ daily activities when considering an PRDP as a treatment option. Other factors should also be considered, such as the remaining number of teeth and tooth loss location, to inform the patients about the potential for future PRDP disuse. Shared decision-making is essential during the pre-treatment phase, involving detailed discussion about the treatment goals and limitations, to ensure patients’ compliance with their PRDP treatment. This study’s results also emphasize the importance for policymakers when considering health coverage plans. Approximately 30% of patients who discontinued using the PRDP were covered by government health insurance. This highlights a potential financial loss that could have been mitigated with more refined treatment screening and planning.

## Conclusion

In this study with up to 7 years follow-up duration, individuals with partial edentulism and sufficient remaining functional dentition without oral impacts were more prone to discontinue PRDP usage. Those with maxillary anterior tooth loss are less likely to discontinue using their PRDPs. The primary reason for initially seeking PRDP treatment was often influenced by the dentists’ suggestion. However, participants reporting oral impacts expressed a clear need for new denture replacements. This highlights the importance of incorporating patient needs and preferences in prosthodontic decision-making.

## Data Availability

The dataset generated during the current study is available upon request to the corresponding author.
